# Commentary: Experiences of being cared-for: the perspective of an expert-by-experience in mental health

**DOI:** 10.3389/fpsyt.2024.1460365

**Published:** 2024-10-04

**Authors:** Hideki Muramatsu

**Affiliations:** Graduate School of Arts and Sciences, The Open University of Japan, Chiba, Japan

**Keywords:** expert-by-experience, autoethnography, caregivers and cared-for, multifamily therapy, paternalism

## 1 Introduction

Fox (1) has created a certain academic value by analyzing and discussing the personal experiences of people with mental ill-health being cared-for and supported by their families, using the research method of autoethnography. However, Fox’s discussion (1) seems to have strong implications for the inorganic consumerism model relationship between service users, caregivers, and professionals. I, like Fox (1), am an expert-by-experience (EBE) (2) with mental ill-health and have been a member of the culture of the mental health welfare field for more than 20 years. This paper is significant because it will lead readers to a new perception of the world by understanding caregiver identity from the perspective of peer support (3), which is inclusive of humanity and not a consumeristic model.

## 2 Caregivers or peers?

As a person who has experienced intense mental health difficulties, I can relate to the relationship that Fox shares with her mother (caregiver) in her Reflective Memories (1). I have experienced chronic crises with severe depression, frequent panic attacks, derangement of ego, and cognitive dysfunction, and I have had the long-term and ongoing support of my devoted mother (caregiver) in my recovery process. Nevertheless, I do not view health and social care practice as a clear dichotomy with a unidirectional power dynamic relationship between “caregivers” and “cared-for” in the consumer model, because as Anthony (4) and Muramatsu (5) have stated, humans are universally in need of personal recovery, and everyone has a mutual support nature as both “caregivers” and “cared-for.” I recognize this in the peer support relationship (3).

Certainly, there are times when a person is in a serious state of mental illness and requires unidirectional support. There was a time when I had extreme anxiety and could not get up all day, except to eat a minimal amount of food, and I received full support. However, over the course of the recovery process, I have developed a mutually supportive relationship with my caregivers. It was also a socially constructed, mutualistic relationship, different in nature compared to a contractual relationship as an inorganic service user in the simple consumer model, that is, an emotional-peer nature as a universal person. We do not live in a mechanical, modern, and scientific world where caregivers provide services to robots and the robots automatically pay money to the caregiver. We are facing a living, flesh-and-blood human who is emotional, irrational, and contradictory, and is subject to the intersubjective influences of dialogue and social structure (6). I do not consider my relationship with my parents and professionals in terms of a one-way power dynamic relationship of “the carer and the cared-for,” or in a contractual or paternalistic sense. I think of it more as the concept of “peer,” a universal humanity of equality that is directly or indirectly related to my life, walking together through the process of personal recovery. It is a humanistic mutual support relationship that facilitates the process of growth through humanistic perception.

For example, in the case of Fox’s Reflection 3 (1), she states that her mother was unable to properly manage her overprotective and intrusive spirit. She was isolated and needed support and help to care in her own right. She thus had very little support in her own right. In some cases, caregivers also have emotional difficulties and ill-health (7). Family caregivers are also more likely to experience social isolation and poor physical and mental health (8, 9). Family dynamics influence individual development and wellbeing, health, illness, and recovery (10). Thus, not only the person requiring care but also their family members, including caregivers, need recovery, as caregiving dynamics are constructed in a relationship that is both complex and ambiguous. To establish truly collaborative support systems and programs, the concept of care must shift from a one-way dynamic power relationship between family members, professionals, and caregivers. Helper therapy principles are essential to the background theory supporting cooperative support systems and programs, and the concept of care is crucial from a whole-system perspective that focuses on the reciprocity of two-way and multiple power dynamic relationships. Not only is receiving unilateral support insufficient to achieve good psychological results (11), but it is also important to reduce the distress caused by receiving support (12). The act of helping others is therapeutic, and providers benefit more in terms of mental wellbeing than the help recipients (13). Reciprocal emotional support is necessary to establish close and supportive bonding and ensure authentic communication, which further leads to improvements in psychological wellbeing (11).

After I went through a major crisis, I used aspects of my recovery process to consciously create a reversed power-dynamic relationship in which I, the caregiver (a different form of caregiving), cared for my mother, leading to my independence, autonomy, and empowerment.

## 3 Conclusion

Focusing on the family, perhaps the elements of the multifamily therapy (14) philosophy, would be appropriate. Rather than interpreting the family as a dualistic “caregiver” and “cared-for,” each member of the family takes on the role of “co-therapists, disorder experts, and agents of change” (15) and each other as peer recognition. Labeling a particular person in the family with a *caregiver identity* can lead to a one-way power dynamic, such as “I will care for you,” from the caregiver to the cared-for person, and in the worst-case scenario, a paternalism or colonialist status quo can occur. Thinking in terms of the unidirectional dynamics of human relations is considered to be at odds with the recent trend toward a new path of a more conscious and connected society characterized by cooperation (16) and the view of the human being that social constructivism opens up (6) with open dialogue (17). Such a status quo may lead to the loss of a cared-for person’s sense of empowerment as well as the caregiver’s personal growth. It may sound paradoxical logic, but learning to care effectively requires driving the paternalistic perspective of “caring effectively” out of one’s consciousness. The concept of the “companion” as an equal partner in the recovery process is important (3), and from the perspective of an EBE (2), I would like to see a shift in the perception of the caregiving relationship in mental healthcare from a consumer model to a more inclusive and humanistic understanding. A humanistic perception of the caregiving relationship in mental healthcare would contribute to the ongoing discussion on mental health recovery and provide insights that can help develop empowering and collaborative support systems.
